# Investigating the potential accessibility to HIV pre-exposure prophylaxis via community pharmacies and sexual health clinics: a scoping review of two integrated care systems in London

**DOI:** 10.1186/s12913-025-12985-2

**Published:** 2025-07-01

**Authors:** Aos Alaa, Datapwa Mujong, Shivali Lakhani, Marsha Alter, Austen El-Osta

**Affiliations:** 1 Self-Care Academic Research Unit, School of Public Health, Imperial College London, White City Campus, 80–92 Wood Lane, London, W12 0BZ United Kingdom; 2 Middlesex Pharmaceutical Group of Local Pharmaceutical Committees, London, United Kingdom

**Keywords:** HIV, PrEP, Community pharmacy, Sexual health clinics, London, Access to care, Public health

## Abstract

**Background:**

Despite advancements in HIV prevention, barriers and disparities in accessing Pre-exposure Prophylaxis (PrEP) from specialist services persist. Community pharmacies, with their extensive reach, offer an opportunity to reduce these disparities and help end new HIV infections.

**Objective:**

This study aims to investigate the accessibility of HIV PrEP through community pharmacies and sexual health clinics (SHCs) across two London Integrated Care Systems (ICS) in the United Kingdom (UK). We also sought to assess the potential for community pharmacies to enhance access to PrEP and to address existing gaps in PrEP provision in the UK to gauge the potential for community pharmacies to facilitate easier access to PrEP.

**Methods:**

We mapped the distribution of pharmacies, PrEP clinics, and SHCs across 11 local authority areas in London, covering North West London ICS and three catchment areas from North Central London ICS using publicly available data, including postcode data. Our selection process for sources of evidence was guided by a set of predefined criteria aimed at identifying community pharmacies and SHCs within the targeted ICSs for their relevance to HIV PrEP accessibility. Two reviewers systematically charted data from each included source. Information extracted included the name of the pharmacy or clinic, address, contact details and operating hours.

**Results:**

Our data collection, spanning from April to July 2022, included 692 community pharmacies. The study revealed that 543 (78.5%) of these pharmacies offered sexual health services, with a significant proportion categorised at Tier 2 according to the Umbrella Sexual Health Model, indicating they are well-positioned to offer PrEP services. The mapping showed an uneven distribution of PrEP and sexual health clinics, with community pharmacies widely accessible and offering longer service hours.

**Conclusion:**

The findings highlight the critical role community pharmacies could play in providing PrEP, addressing gaps in accessibility, and reducing HIV transmission risks, thus potentially facilitating easier access to PrEP. This study supports the need for policy adjustments to enable community pharmacies to dispense PrEP, aligning with public health goals for broader access to HIV preventative measures.

## Introduction

HIV Pre-exposure prophylaxis (PrEP) is recommended by the World Health Organization (WHO) for individuals at substantial risk of HIV infection, available in oral, intramuscular, and vaginal ring formulations [1, 2]. While highly effective, the delivery of PrEP has primarily benefited men who have sex with men and white populations, leaving other at-risk groups less served [1–3].

Despite significant improvements across England, London continues to have the highest rate of new HIV diagnoses in the country. Globally, new HIV infections have declined to their lowest level since 2016 [4], the same year NHS England initially refused to fund PrEP, yet 1.5 million new infections were still recorded in 2021 [5]. North West London (NWL) is an area of concern with high to very high prevalence of HIV [6]. The London Borough of Hammersmith and Fulham in NWL Integrated Care System has one of the highest HIV diagnosis prevalence in United Kingdom of 7.7/1,000 people aged 15–59 highlighting the urgent need for effective prevention strategies [7]. Data from the UK Health Security Agency (UKHSA) and the London HIV Epidemiological Spotlight (2021) indicate that boroughs within North West London [3] and North Central London [6] ICSs, and particularly Brent, Hounslow, Haringey and Westminster, have high HIV prevalence rates among ethnically diverse and economically marginalised populations [4–7]. Brent and Haringey specifically report a high overall HIV prevalence coupled to substantial rates of late diagnosis and lower levels of HIV testing uptake among Black and minority ethnic groups.

The high to extremely high HIV prevalence rates across all eight boroughs in NWL presents a unique opportunity for broadening PrEP access due to its dense network of pharmacies and sexual health clinics (SHCs) [8]. Following significant advocacy efforts, the UK’s National Health Service (NHS) began offering PrEP in 2017, with a significant policy announcement in 2020 expanding free PrEP access to high-risk individuals, marking a pivotal moment in the fight against HIV [9, 10]. However, the challenge remains to extend PrEP access beyond sexual health services to ensure equity for all communities at risk.

The rollout of PrEP in England has begun in specialist sexual health services [11], whereas community-oriented approaches to PrEP delivery are gaining traction, exploring the potential of primary care, community health workers and digital health platforms can reduce access disparities. Pharmacies, integrated within communities and often providing sexual health services, represent an underexplored avenue for PrEP distribution. They offer the advantage of being less stigmatised, accessible during extended hours, and capable of providing personalised care, which could significantly improve PrEP adherence and patient outcomes [12–14].

The feasibility and acceptability of pharmacy-based PrEP distribution have been demonstrated internationally [15–21], yet evidence from the UK remains sparse. Pharmacies are well-positioned for integrating PrEP into their services, offering testing and linkage to care, adherence counselling and leveraging their extended hours and community trust. Pharmacists could be instrumental in educating patients about PrEP, addressing non-adherence and ensuring patients receive comprehensive care, making pharmacists crucial members of the HIV prevention team. This includes engagement with men who have sex with men (MSM), Black African and Caribbean communities, and individuals from lower socio-economic backgrounds who are disproportionately affected by HIV in London.

Although NHS PrEP is widely available through specialist SHCs, these services are often underutilised by communities most at risk due to barriers such as stigma, limited awareness, inconvenient locations, or restricted hours of operation [8, 9]. Few studies have systematically mapped the availability, distribution and service capacity of community pharmacies relative to existing PrEP and sexual health clinic infrastructure. Siegler et al. illustrated a formal Geographic Information Systems (GIS) analysis of PrEP accessibility, offering a detailed examination of geographic access to PrEP clinics in the U.S. context [22], detailing the complexities of integrating PrEP services into pharmacies [22]. There is limited research examining the distribution of PrEP services across different service providers such as SHCs and community pharmacies in the UK context. This absence of localised data constrains both strategic planning and service innovation aimed at widening PrEP access beyond specialist settings. Our study addresses this critical knowledge gap by conducting a scoping review and geospatial analysis of community pharmacies, sexual health clinics and PrEP provision across two London Integrated Care Systems. By examining service availability, tiered sexual health competence and spatial distribution, we generate evidence on where, how, and to what extent community pharmacies could serve as decentralised, accessible points for PrEP delivery.

The primary aim of this study was to investigate the distribution of HIV PrEP clinics, SHCs and community pharmacies within two ICSs in London. We also sought to assess the potential of community pharmacies to make PrEP more accessible and task shift their provision of PrEP.

## Methods

This study aimed to map all pharmacies, SHCs and PrEP clinics across 11 Local Authorities in London. We used publicly available data to identify and map pharmacies across 11 local authority (LA) catchment areas in London spanning two ICSs; NWL ICS and three catchment areas from NCL [23] ICS. Postcode data was used to map the distribution of pharmacies, PrEP clinics and SHCs on a map. This scoping review was not aided by a review protocol.

### Protocol and registration

No protocol was preregistered. This review was part of a larger programme of work that received a favourable opinion from the Imperial College London Research Ethics Committee (#21IC6934).

### Eligibility criteria

The included pharmacies, SHCs, and PrEP clinics were located within the catchment areas of the NWL ICS or three designated catchment areas from the NCL ICS. We included only those operational pharmacies and clinics that provided services to the public during our data collection period from 30 April 2022 to 4 July 2022. For community pharmacies, inclusion required the provision of any sexual health services, as this indicated potential for PrEP distribution. For sexual health clinics, inclusion necessitated offering PrEP services or consultations, aligning with our focus on PrEP access points. Entities that ceased operation before or during our study period were excluded to maintain the relevance and accuracy of our data. Additionally, pharmacies and clinics outside the specified ICS catchment areas were excluded to adhere to our geographical focus.

### Information sources

We leveraged our personal and professional contacts to compile a list of community pharmacies from 11 LA catchment areas in London (eight LAs in NWL (Brent, Ealing, Hammersmith and Fulham, Harrow, Hillingdon, Hounslow, Kensington and Chelsea, and Westminster) and three LAs (Barnet, Enfield and Haringey) in NCL ICS). This list was obtained and updated in April 2022 and includes each pharmacy’s Organisation Data Service (ODS) code, pharmacy name, region, address and contact details (including telephone number). We also obtained a list of all SHCs and PrEP clinics across London. We obtained a list of SHCs across NWL and PrEP clinics across London through the NHS Digital website on 11 April 2022. This dataset included the clinic name, address, postcode and telephone number. We conducted thorough searches of public health databases and official NHS websites, including the NHS Digital and the Sexual Health London service. Specifically for community pharmacies, we utilised NHS’ online ‘Find a Pharmacy’ tool [24] to help us identify pharmacies within our target ICS catchment areas. This Find a Pharmacy tool details the services provided at each pharmacy (e.g. contraception services, healthy living services, prescription services, screening and test services) and information on service offerings that are relevant to PrEP provision (e.g. sexual health consultations and vaccinations requiring injection). Where necessary, we directly contacted pharmacies and clinics to verify service offerings and operational status. This step was crucial for ensuring the accuracy of our data, particularly for services not fully detailed in public listings or where recent changes may not have been reflected.

### Search strategy

Our search strategy was designed to ensure comprehensive coverage and retrieval of data regarding community pharmacies and SHCs within the targeted London ICSs. This strategy aimed to map the current landscape of HIV PrEP availability, focusing on identifying all potential access points for the communities served by these ICSs.

Initially, we developed a set of keywords relevant to our study objectives, including “HIV PrEP”, “community pharmacy”, “sexual health services”, and “Pre-exposure prophylaxis.” These keywords were used to ensure that our search would capture the broadest possible range of relevant services. We also conducted searches on through NHS Digital and the Sexual Health London website [25]. The search was conducted within a defined period, from 30 May 2022 to 4 July 2022. Specifically for pharmacies, we utilised the NHS’s ‘Find a Pharmacy’ online service, inputting our keywords and focusing on the geographic areas of interest. This tool provided detailed information on pharmacy services, including whether SHCs offered PrEP-related services.

Three medical students, one researcher and one Masters in Public Health (MPH) student obtained a list of services provided by each pharmacy in NWL and NCL using the pharmacy search function on the NHS website [24]. To supplement our online searches and ensure the accuracy of our findings, we directly contacted a subset of pharmacies and clinics. This step involved verifying service offerings and operational status, particularly for entities with ambiguous or outdated online information.

### Selection of sources for evidence

Our selection process for sources of evidence was guided by a set of predefined criteria aimed at identifying community pharmacies and SHCs within the targeted ICSs for their relevance to HIV PrEP accessibility. Following the comprehensive search, we compiled an extensive list of potential sources, including community pharmacies and sexual health clinics. Each source was initially screened based on its geographical location to ensure it fell within the NWL London ICS or the specified areas of the NCL ICS. Sources that passed the initial screening underwent a detailed review to ascertain their offerings related to sexual health services and specifically, PrEP provision. This involved analysing the information gathered from public databases, the NHS ‘Find a Pharmacy’ tool [24], and direct contact verifications to ensure that each source met our criteria. We included organisations that provided sexual health services, with a specific focus on those offering PrEP consultations, dispensing, or both. This included pharmacies with Tier 1 (T1) or Tier 2 (T2) services according to the Umbrella Sexual Health Model [26] and SHCs offering PrEP services. We excluded pharmacies and clinics that did not offer sexual health services, were not operational during our data collection period, or were located outside the specified catchment areas.

The selection process was collaborative, involving five reviewers (AA, DM, LK, MM, IC) to mitigate bias and ensure consistency. Discrepancies between reviewers regarding the inclusion or exclusion of sources were resolved through discussion, and if necessary, consultation with a third-party adjudicator.

### Data charting process and data items

Prior to charting, we developed a detailed data extraction template in Microsoft Excel. This template was designed to capture both quantitative and qualitative data relevant to our study objectives, ensuring consistency across reviewers in the data charting process. All team members involved in data charting underwent training to familiarise themselves with the extraction template and to ensure consistent understanding and application of the data extraction criteria.

Using the prepared template, reviewers systematically charted data from each included source. Information extracted included the name of the pharmacy or clinic, address, contact details and operating hours. In terms of service offerings, we included details on sexual health services provided, with a focus on PrEP-related services such as consultations, dispensing and any additional support services offered. Data charted by individual reviewers were cross-checked by a second reviewer to verify accuracy and completeness. Discrepancies were resolved through discussion or, if needed, re-examination of the source material. This information was extracted using the NHS Find a Pharmacy tool [24]. Pharmacies were categorised based on their rating as either Tier 0, 1 or 2 (as per Table 1).

**Table 1:** Asset rating characteristics derived for rating pharmacy competence

Rating	Tier 0	Tier 1	Tier 2
Asset rating description	• No sexual health service offered	• Emergency hormonal contraception (EHC) • Chlamydia screening (with EHC) • STI kit “click and collect” service • Condoms	• All Tier 1 services • Oral contraception– start up • Oral contraception– continuation • Injectable contraception • STI kit initiation • Chlamydia treatment • Hepatitis B vaccination (2nd & 3rd dose)
Notes	• Pharmacy not engaged in any sexual health provision • May refer to other providers	• Basic sexual health support available • Often commissioned under local public health contracts	• Enhanced sexual health service delivery • Requires PGDs for medicines • Staff must complete accredited training (e.g., CPPE modules) • May support sexual health promotion campaigns • Local authority/NHS with PGDs
Commissioning status	• Not commissioned	• Local authority commissioned or NHS-funded	
Training/accreditation requirements	• None	• Basic safeguarding training; awareness of EHC protocols	• CPPE sexual health e-learning; PGD compliance; clinical supervision

*Abbreviations*: *EHC* Emergency hormonal contraception, *STI* Sexually transmitted infection, *CPPE* Clinical pharmacists in general practice; duration, *PGD* Patient group directions

### Critical appraisal of individual sources of evidence

To quality assess each source, we developed a set of criteria tailored to the specific context of our study. These criteria included the comprehensiveness of service offerings related to PrEP, the accuracy and currency of the information provided, and the operational status during the study period. Given the diversity and variability of the sources of evidence used in our study, we implemented a critical appraisal process to assess the quality and relevance of each selected community pharmacy and sexual health clinic. Using the Umbrella Sexual Health’s model [26], we adapted the gov.uk “tier” system [27] for pharmacies and developed our own “level” system to categorise the pharmacies based on the sexual health services they provided. We used the Umbrella Sexual Health Model’s tier system (T0, T1, or T2) to classify pharmacies based on the range of sexual health services provided. Information on the physical accessibility of the location, availability of private consultation areas, and any language support services offered were also recorded.

### Synthesis of results

Extracted data were synthesised using tallies and percentages to describe the number of pharmacies offering sexual health services per LA catchment area. We aggregated the data extracted from individual sources, categorising them according to key variables of interest such as geographic location, type of service provider (community pharmacy vs. sexual health clinic) and the range of PrEP-related services offered.

The postcode was used on Google’s “My Maps” tool to illustrate the spatial distribution of pharmacies, SHCs and PrEP clinics across both Integrated Care Systems. This freely accessible visualisation platform was selected as it can support preliminary spatial mapping, although it lacks the functionality and customisation capabilities of full GIS software. The resulting map distinguished between different service providers, the services offered (e.g., SHS, Vaccinations and whether the clinic has a private consultation room), the availability of PrEP services during extended hours, and the representation of Tier 0, 1 or 2 pharmacies in the overall service landscape. Each asset type was assigned a unique colour label. This approach helped us identify patterns and trends in the provision of SHS and PrEP services across the targeted ICSs.

A critical component of our synthesis involved identifying gaps in PrEP accessibility, particularly for minority groups and in densely populated urban areas. By comparing the distribution of services against population density and demographic data, we pinpointed areas where service enhancements could significantly impact PrEP accessibility. Finally, we conducted a comparative analysis between the two ICSs to assess variations in service provision and identify best practices and areas for improvement. This comparison offered valuable insights into how different organizational and operational models impact PrEP service accessibility.

### Ethics

Not applicable. This review was conducted as part of a larger programme of work, which received ethical approval by Imperial College Research Ethics Committee (ICREC #21IC6934).

### Patient and public involvement

No patient was involved.

## Results

### Selection of sources of evidence

**Fig. 1:**
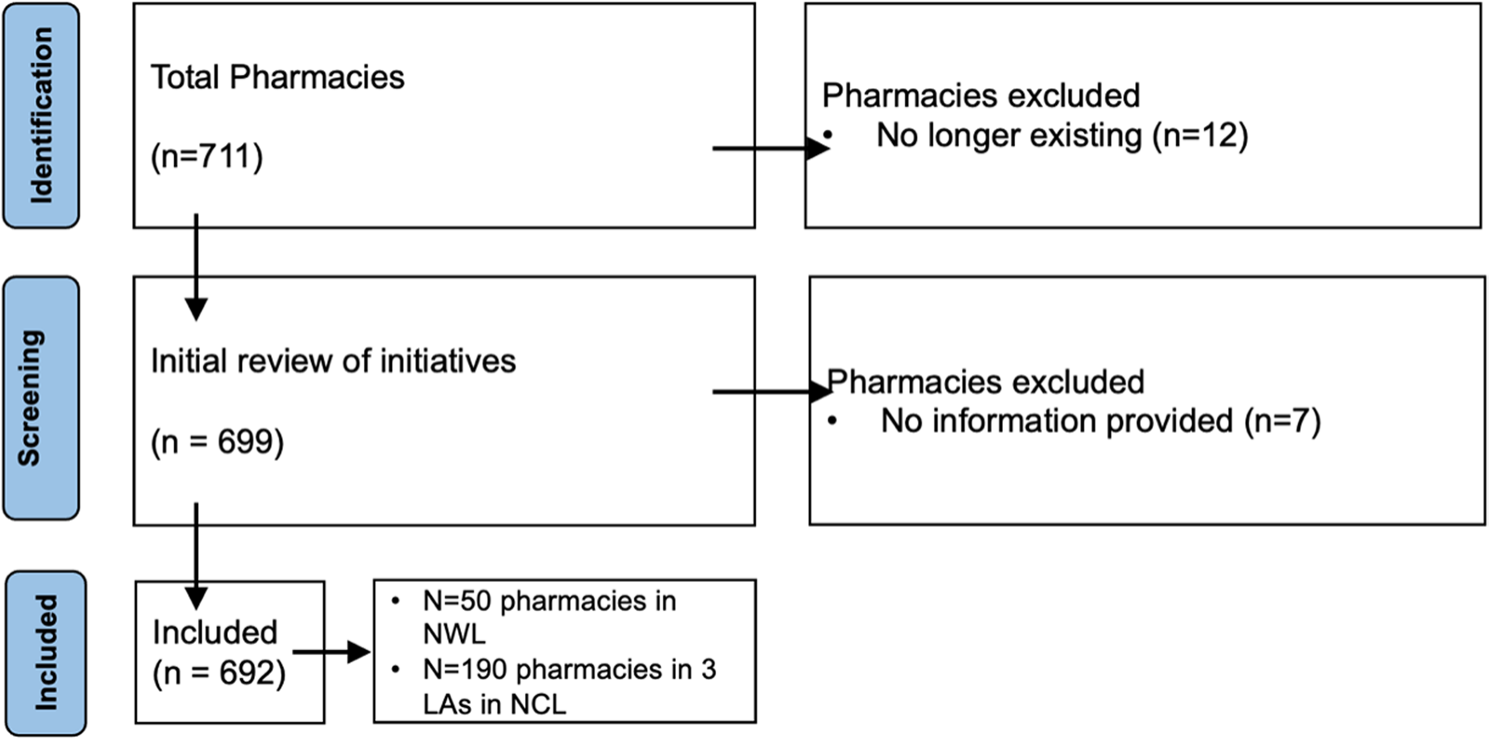
PRISMA flowchart showing the process of excluding pharmacies

Through personal and professional contacts, we were able to identify 711 community pharmacies, of which 19 were excluded because they were no longer open for business. Figure 1 provides the PRISMA flowchart detailing the selection process. A total of 692 community pharmacies were analysed: 502 pharmacies in NWL and 190 pharmacies from 3 LAs in NCL (Table 2).

**Table 2:** Place-based assessment of community pharmacy landscape in 11 LAs in London

ICS	Local Authority	Population (2022)*	No. Pharmacies	No. pharmacies Per 10,000 people	Sexual Health Clinics	PrEP Clinics	Offers SHS			Tier				Offers Vaccinations	Has private consultation room^**^
							Yes	No	Unknown	0	1	2	Unknown		
					*N* (%)	*N* (%)	*N* (%)	*N* (%)	*N* (%)	*N* (%)	*N* (%)	*N* (%)	*N* (%)	*N* (%)	*N* (%)
NWL	Brent	341,221	81	2	0 (0.0)	1 (16.7)	69 (85.2)	4 (4.9)	8 (9.9)	4 (4.9)	62 (76.5)	7 (8.6)	8 (9.9)	60 (74.1)	70 (86.4)
	Ealing	369,973	76	2	1 (16.7)	0 (0.0)	64 (84.2)	9 (11.8)	3 (3.9)	9 (11.8)	59 (77.6)	5 (6.6)	3 (3.9)	64 (84.2)	70 (92.1)
	Hammersmith & Fulham	185,238	40	2	4 (66.7)	1 (16.7)	27 (67.5)	11 (27.5)	2 (5.0)	11 (27.5)	24 (60.0)	3 (7.5)	2 (5.0)	37 (92.5)	36 (90.0)
	Harrow	261,185	64	2	0 (0.0)	0 (0.0)	44 (68.8)	10 (15.6)	10 (15.6)	10 (15.6)	37 (57.8)	7 (10.9)	10 (15.6)	49 (76.6)	53 (82.8)
	Hillingdon	310,681	64	2	0 (0.0)	0 (0.0)	55 (85.9)	8 (12.5)	1 (1.6)	8 (12.5)	45 (70.3)	10 (15.6)	1 (1.6)	57 (89.1)	58 (90.6)
	Hounslow	290,488	53	2	1 (16.7)	1 (16.7)	41 (77.4)	9 (17.0)	3 (5.7)	9 (17.0)	26 (49.1)	15 (28.3)	3 (5.7)	42 (79.2)	45 (84.9)
	Westminster	211,365	84	4	0 (0.0)	2 (33.3)	58 (69.0)	14 (16.7)	12 (14.3)	15 (17.9)	52 (61.9)	6 (7.1)	11 (13.1)	61 (72.6)	66 (78.6)
	Kensington & Chelsea	146,154	40	3	0 (0.0)	1 (16.7)	31 (77.5)	7 (17.5)	2 (5.0)	7 (17.5)	27 (67.5)	4 (10.0)	2 (5.0)	35 (87.5)	36 (90.0)
	All NWL	2,116,305	502	2	6 (100.0)	6 (100.0)	389(77.5)	72 (14.3)	41 (8.2)	73 (14.5)	332 (66.1)	57(11.4)	40 (8.0)	405 (80.7)	424 (84.5)
NCL	Barnet	389,101	76	2	2 (50.0)	1 (33.3)	61 (80.3)	12 (15.8)	3 (3.9)	12 (15.8)	53 (69.7)	7 (9.2)	4 (5.3)	65 (85.5)	72 (94.7)
	Enfield	327,224	57	2	1 (25.0)	1 (33.3)	45 (78.9)	9 (15.8)	3 (5.3)	9 (15.8)	38 (66.7)	9 (15.8)	1 (1.8)	47 (82.5)	52 (91.2)
	Haringey	261,811	57	2	1 (25.0)	1 (33.3)	48 (84.2)	6 (10.5)	3 (5.3)	6 (10.5)	25 (43.9)	22 (38.6)	4 (7.0)	44 (77.2)	51 (89.5)
	All 3 NCL	978,136	190	2	4 (100.0)	3 (100.0)	154(81.1)	27 (14.2)	9 (4.7)	27 (14.2)	116 (61.1)	38 (20.0)	9 (4.7)	156 (82.1)	175 (92.1)
Total		3,094,441	692	2	10 (100.0)	9 (100.0)	543 (78.5)	99 (14.3)	50 (7.2)	100 (14.5)	448 (64.7)	95 (13.7)	49 (7.1)	561 (81.0)	609 (87.9)

^*^Population data from Office of National Statistics [28]

^**^Private consultation room– this is a room in the clinic where patients can discuss sensitive topics with their pharmacist in privacy

### Critical appraisal within sources of evidence

#### Characteristics of pharmacies according to tier

Over three quarter of these pharmacies 543 (78.5%) offered sexual health services. Of these pharmacies, 100 (15.5%) were T0, 448 (69.6%) were T1, and 95 (14.9%) were T2 (coinciding with the Umbrella sexual Health Model) (Table 2).

It was not possible to identify whether 51 (7.4%) of these pharmacies offered sexual health services and were therefore unable to categorise these pharmacies into a tier. 561 (81.0%) of pharmacies offered vaccination services, and 609 (87.9%) of these pharmacies had a private consultation room on site; Table 2.

#### Characteristics of PrEP and sexual health clinics across 11 local authorities in London

From NWL, we identified six total SHCs and six PrEP Clinics, some of which overlap (Table 2). Of the sexual health clinics, four (66.7%) of these are found in Hammersmith and Fulham, one in Ealing (16.7%) and one in Hounslow (16.7%). And of the PrEP clinics, two are situated in Westminster (33.3%), one (16.7%) in Kensington and Chelsea, one (16.7%) in Hammersmith and Fulham, one (16.7%) in Hounslow, and one (16.7%) in Brent; Table 2.

### Results of individual sources of evidence

Of all the pharmacies in NWL and 3 LAs in NCL (*n* = 692), 543 (78.5%) offer sexual health services, 95 (14.9%) were categorised at T2 pharmacies, 561 (81.0%) offer vaccinations, and 609 (87.9%) have a private consultation room. When comparing the proportion of T2 pharmacies between NWL and 3 LAs from NCL, only 57 (11.4%) of pharmacies in NWL were T2, whereas 38 (20.0%) of pharmacies in NCL were tier 2.

### Synthesis of results

**Fig. 2:**
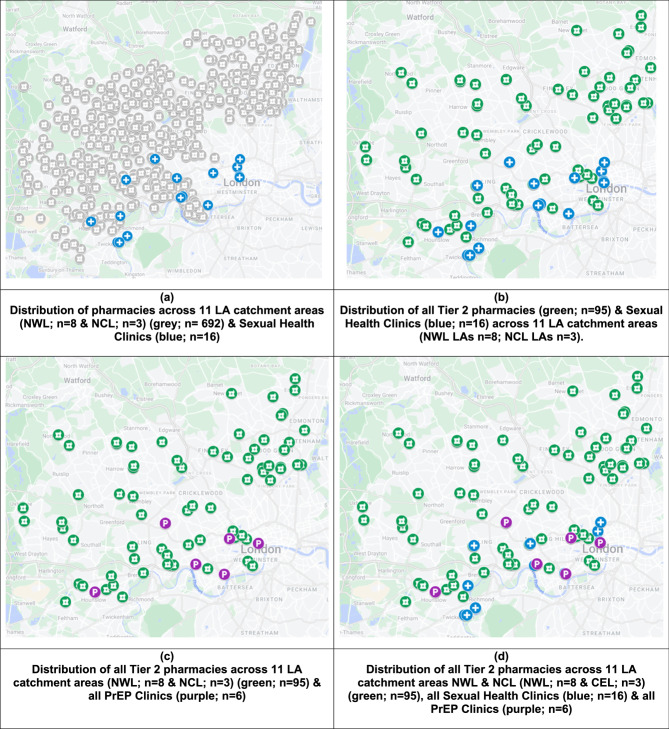
The distribution of pharmacies with respect to their tiers, sexual health clinics and PrEP clinics across NWL and 3 LAs in NCL

To synthesise the results, we mapped of PrEP and SHCs Across 11 Local Authorities in London. Figure 2a shows the distribution of SHCs (*n* = 16) across all 8 local authorities in NWL and 3 local authorities in NCL. Figure 2b shows the distribution of all T2 pharmacies (*n* = 95) and SHCs (*n* = 16) in NWL and 3 LAs in NCL. Figure 2c shows all T2 pharmacies (*n* = 97) and PrEP clinics (*n* = 6) in NWL and 3 LAs in NCL. Figure 2d shows all the T2 pharmacies (*n* = 95), all SHCs (*n* = 16) and all PrEP clinics (*n* = 6) in NWL and 3 LAs in NCL.

### Availability of consultation rooms

The vast majority of pharmacies (89.7% in all our LAs, 84.5% in NWL LAs) had private consultation rooms which could be used to supports confidential and discreet access to PrEP.

## Discussion

### Summary of evidence

Our comprehensive study across NWL and three boroughs of NCL revealed that a substantial majority of community pharmacies *n* = 543 (79%) provide sexual health services, with a notable portion (13.7%) equipped to offer Tier 2 services, encompassing a broad range of sexual health interventions. Despite this, we observed an uneven distribution of SHCs and PrEP services across NWL and NCL, highlighting potential access gaps, with two thirds of all SHCs in NWL being located in Hammersmith and Fulham, while half of the NCL SHCs are in Barnet. Community pharmacies, especially those with Tier 2 capabilities, emerged as potentially pivotal in widening access to PrEP.

The integration of community pharmacies into the HIV prevention framework highlights the untapped potential to decentralise PrEP distribution, enhancing service accessibility. This approach aligns with global public health goals, aiming to bridge disparities in PrEP access and reduce new HIV infections [29]. Our findings add to the growing evidence base, supporting pharmacy-based models for PrEP distribution as feasible and acceptable. Notably, community pharmacies in urban settings like London are well-positioned to significantly contribute to public health goals, addressing high HIV prevalence and reaching traditionally underserved communities.

Our data indicated that a majority (81%) of pharmacies offered vaccination services, suggesting an existing infrastructure that could potentially be leveraged for the delivery of injectable PrEP subject to regulatory and training considerations. Additionally, the presence of private consultation rooms in 88% of pharmacies highlights the potential for confidential PrEP consultations and services, an essential component of effective sexual health service delivery. This highlights community pharmacies as key to bridging PrEP access gaps, pinpointing areas for targeted intervention within London’s Integrated Care Systems. Furthermore, The convenience offered by community pharmacies, typically within a 15-minute walk for urban populations, alongside their longer operating hours compared to other healthcare providers [30], marks them as a vital resource for PrEP accessibility.

### Comparison to existing literature

This study is the first of its kind to analyse the distribution of services in LAs. Our study highlights the potential viability and acceptance of pharmacy-based PrEP distribution noted in existing literature while introducing new insights into accessibility challenges within London’s urban environment. It highlights the importance of addressing logistical and regulatory hurdles to enhance PrEP access, advocating for culturally competent healthcare solutions.

Echoing findings from Kennedy et al. [20], we emphasise community pharmacies’ potential role in providing accessible, stigma-free PrEP services, crucial for reaching marginalised populations. Our analysis of London’s community pharmacies reveals a strong readiness to participate in sexual health services, which is crucial in addressing ongoing HIV transmission challenges and reaching vulnerable groups more effectively. By mapping the distribution of pharmacies relative to PrEP and sexual health clinics, we offer a localised view of accessibility, similar to Raifman et al. (2020)’s [31] spatial analysis in the U.S.

Our review revealed geographical disparities in service provision. For instance, while certain LAs like Hammersmith and Fulham and Westminster showcased a higher concentration of pharmacies providing Tier 2 services, other areas exhibited gaps that could potentially limit access to PrEP and other critical sexual health services for local populations. Other local authorities like Haringey have 23 registered pharmacies providing higher level service including 12 that offer HIV testing, but this augmented offer appears to be lacking in other local authorities highlighting both a gap and a potential for leveraging pharmacies. Collectively, these findings make the need for targeted interventions to enhance service availability, especially in underserved areas, and highlights the strategic role community pharmacies could play in broadening access to PrEP across diverse communities. Our study findings align with Calabrese et al. [32] on the need for healthcare interventions to be tailored to underserved communities, highlighting community pharmacies’ potential to provide discreet, culturally sensitive services.

### Study implications

The implications of our study for public health policy and practice are significant, highlighting the potential role of community pharmacies in HIV prevention. For a HIV-free generation, integrating pharmacies into the national HIV prevention strategy is essential, leveraging their accessibility to mitigate PrEP access disparities, especially for marginalised communities. This integration requires policy reforms for seamless PrEP distribution and ensuring pharmacies have the requisite training and resources. Community pharmacies present an accessible PrEP option, potentially easing healthcare system burdens. Effective service delivery hinges on comprehensive pharmacist training in PrEP counselling and management. Furthermore, pharmacies should act as community hubs for raising PrEP awareness, necessitating collaboration with public health entities and community organisations to enhance service uptake.

Their strategic locations and extended hours offer a less stigmatising and more accessible option for obtaining PrEP, a crucial aspect for BAME and Somali populations, which face barriers in traditional sexual health services access [33]. In future, studies we would like to explore methods to objectively quantify the distance travelled to each PrEP clinic per borough, this way we can better target specific boroughs with barriers to access.

Our findings advocate for further research into pharmacy-based PrEP models to understand their implementation and impact better. Our findings show that a significant proportion of pharmacies in the 11 LAs, 543 (78%), offered sexual health services, indicating a strong potential for community pharmacies to play a pivotal role in enhancing access to PrEP. The vast majority of pharmacies (90% across the Local Authorities included) had private consultation rooms which could be used to supports confidential and discreet access to PrEP. Mitigating stigma and facilitating easier engagement for ethnic minority groups. Furthermore, the high level of training and skills among pharmacists, evident in their provision of advanced services, for example offering injectable contraception, highlights their capacity to deliver PrEP effectively.

By making community pharmacies central to HIV prevention, we aim for a more inclusive approach to healthcare, crucial for public health advancements and achieving an HIV-free future. Coordinated efforts from all healthcare stakeholders are vital to harnessing the full potential of community pharmacies, guided by evidence and a commitment to healthcare accessibility and equity.

### Strengths and limitations

Our study’s strength lies in the comprehensive mapping of community pharmacies and SHCs across two London Integrated Care Systems, offering a detailed panorama of PrEP accessibility in an urban context. The principal limitation of this study is the specific focus on London’s unique urban environment and healthcare infrastructure, which means that our results may not be directly transferrable to other regions with different socioeconomic conditions and healthcare systems. The reliance on publicly available data, while comprehensive, introduces a limitation as such data may not capture the most current practices or the entirety of services offered by pharmacies and clinics, potentially affecting the accuracy and representability of our findings. Additionally, the absence of direct input from PrEP users limits our understanding of accessibility and service utility from the community’s perspective, a gap that future studies could address through qualitative research methods. Operational challenges related to the implementation of pharmacy-based PrEP distribution, such as training needs, privacy concerns, and regulatory navigation were not fully explored, highlighting the need for an in-depth analysis of these barriers in subsequent research. Lastly, the observational nature of this scoping review study precludes the establishment of causality between pharmacy service provision and improved PrEP accessibility. Future research could involve longitudinal or intervention-based studies as these will offer a more nuanced understanding of the dynamics at play in the effective distribution of PrEP and the role of community pharmacies therein.

While our study highlights borough-level HIV prevalence and maps the distribution of potential PrEP access points, a key limitation in the lack of stratified epidemiological data linking specific underserved populations. As such, while our findings suggest that community pharmacies are well-positioned to serve ethnically diverse and potentially underserved populations in boroughs like Brent, Haringey and Hounslow, we caution against overgeneralisation. The geographies of HIV risk among different minoritised groups are likely heterogeneous and require dedicated investigation using disaggregated ethnicity-incidence data and qualitative inquiry into access barriers. Future research should incorporate finer-grained sociodemographic and behavioural data to better align pharmacy-based PrEP delivery strategies with the nuanced needs of specific populations within each Integrated Care System.

Another limitation is related to the use of the “My Maps” served as a pragmatic tool to visually communicate the distribution and density of service providers. As this was not a formal GIS, did not seek to conduct advanced spatial analysis or to estimate the time and travel costs based on distance.

Future research should aim to evaluate the implementation and impact of PrEP distribution through community pharmacies, focusing on effectiveness in diverse settings and identifying operational barriers. Longitudinal and comparative studies across various healthcare contexts are essential to ascertain the best models for PrEP delivery that are accessible, cost-effective and patient-oriented. Additionally, qualitative research into the experiences of high-risk groups will provide insights into overcoming stigma and enhancing service uptake. Understanding these dynamics is crucial for developing tailored, culturally sensitive healthcare interventions that can make PrEP more accessible and acceptable to all communities.

## Conclusion

Our study highlights vast distribution and capabilities of community pharmacies in comparison to SHCs and guides the significant role which community pharmacies can play in improving access to HIV PrEP in London, demonstrating their potential to fill critical gaps in PrEP provision. Emphasising the necessity for policy reforms and targeted training, this approach aligns with global health goals to combat HIV. Realizing the full potential of community pharmacies in HIV prevention requires collaborative efforts across the healthcare system, ensuring PrEP accessibility and equity. Future work must focus on overcoming implementation barriers, paving the way for community pharmacies to become integral to a comprehensive and effective HIV prevention strategy.

## Data Availability

The data that support the findings of this study are available from the corresponding author, AA, upon reasonable request.
